# Excision of staphylococcal cassette chromosome *mec* in methicillin-resistant *Staphylococcus aureus* assessed by quantitative PCR

**DOI:** 10.1186/s13104-015-1815-3

**Published:** 2015-12-29

**Authors:** Miloš Stojanov, P. Moreillon, O. Sakwinska

**Affiliations:** Department of Fundamental Microbiology, University of Lausanne, Lausanne, Switzerland; Institute of Microbiology, University Hospital of Lausanne, Lausanne, Switzerland; Nestlé Research Center, Lausanne, Switzerland

**Keywords:** SCC*mec*, Excision, MRSA, *Staphylococcus aureus*

## Abstract

**Background:**

Methicillin-resistance in staphylococci is conferred by the *mecA* gene, located on the genomic island Staphylococcal Cassette Chromosome *mec* (SCC*mec*). SCC*mec* mobility relies on the Ccr recombinases, which catalyze insertion and excision form the host’s chromosome. Although being a crucial step in its horizontal transfer, little is known about the dynamics of SCC*mec* excision.

**Results:**

A quantitative PCR-based method was used to measure the rate of SCC*mec* excision by amplifying the chromosome–chromosome junction and the circularized SCC*mec* resulting from excision. SCC*mec* excision rate was measured in methicillin-resistant *Staphylococcus aureus* (MRSA) strain N315 at various growth times in broth cultures. In the present experimental settings, excision of SCC*mec* occurred at a rate of approximately 2 × 10^−6^ in MRSA N315.

**Conclusion:**

This work brings new insights in the poorly understood SCC*mec* excision process. The results presented herein suggest a model in which excision occurs during a limited period of time at the early stages of growth.

**Electronic supplementary material:**

The online version of this article (doi:10.1186/s13104-015-1815-3) contains supplementary material, which is available to authorized users.

## Background

*Staphylococcus aureus* is a successful human pathogen, causing a large variety of diseases that range from minor skin infections to life-threatening bloodstream infections and endocarditis [1]. Many of the virulence factors encoded by *S. aureus* are located within mobile genetic elements, which come in addition to the well-conserved core genome, and account for the majority of genetic variation between the isolates [2–4]. This is also often the case for antibiotic resistance genes, which can be found on plasmids, transposons or genomic islands [4].

Methicillin-resistant *S. aureus* (MRSA) arose during the 1960s, due to the acquisition of the Staphylococcal Cassette Chromosome *mec* (SCC*mec*) [5]. SCC*mec* is a staphylococcal genomic island carrying the *mecA* gene, which encodes the low affinity penicillin-binding protein 2a (PBP2a) that is responsible for cross resistance to virtually all antibiotics of the β-lactam family [5, 6]. It is inserted at a unique location in the chromosome by the Ccr proteins (cassette chromosome recombinases), which catalyze site-specific recombination between a 3′ end of the *rlmH* gene (*attB*), previously known as *orfX*, and the homologous sequence on SCC*mec* (*attS*). Upon integration the *attS* and the *attB* sequences form two direct repeats (designated *attL* and *attR*) flanking the cassette [7, 8]. In the opposite path, Ccr proteins catalyze the excision of SCC*mec* from the host’s chromosome [8, 9].

Early clues of SCC*mec* excision rate were reported by Ito et al., who detected excision of the cassette in MRSA N315 at a rate of less than 10^−4^ [10]. Nevertheless, precise excision rate and dynamics of SCC*mec* excision have never been determined. To address this issue, we used a qPCR-based system allowing us to quantify precisely the rate of SCC*mec* net excision from the chromosome of MRSA strain N315, as well as to detect the resulting excised closed circular forms appearing in the cell.

## Methods

### Bacterial strains, media and culture conditions

Bacterial strains and plasmids used in this study are listed in Table 1. *Escherichia coli* strain DH5α was routinely used for plasmid propagation and cloning experiments, and cultivated on Luria-Bertani (LB) medium (Becton–Dickinson, Sparks, MD, USA) supplemented with 100 mg/L ampicillin (AppliChem, Darmstadt, Germany) at 37 °C. *S. aureus* strains were grown with aeration in trypticase soy broth (TSB) (Difco Laboratories, Detroit, MI, USA) in a rotating incubator (at 180 rpm) at 37 °C. If required, tetracycline and kanamycin (AppliChem) were added at a final concentration of 10 mg/L. Oxacillin (Sigma-Aldrich, Buchs, Switzerland) was used at 4 mg/L, which corresponded to the MICs for strain N315.

**Table 1:** Bacterial strains and plasmids

Strain or plasmid	Relevant characteristics	References
Strains		
*E. coli* DH5α	Host for DNA cloning	Laboratory collection
*S. aureus*		
RN4220	Restriction-deficient derivative of *S. aureus* RN450	[11]
N315	MRSA carrying type II SCC*mec*	[12]
N315EX	Isogenic MSSA derivative of N315	This study
Plasmids		
pUC28	ColE1 replicon, high copy number vector for cloning, ampicillin resistance	[13]
pSR3-1	Thermosensitive-replicon plasmid carrying the *ccrAB* genes of strain N315 (used for SCC*mec* excision in N315), tetracycline resistance	[7]
qPlasmid	Plasmid used for qPCR analysis	This study

### DNA manipulations

Genomic DNA of *S. aureus* was extracted using a protocol described previously [14]. For genomic DNA, 3 mL of overnight cultures were harvested and resuspended in 50 μL of lysis solution (0.5 μg/mL lysostaphin, 10 mM Tris–Cl, pH 7.5, and 1 mM EDTA). After 30 min of incubation at 37 °C, cell suspension were lysed by adding 300 μL of “Nuclei Lysis Solution” (Promega Corporation, Madison, WI) and heating at 80 °C for 10 min. After RNase treatment, 100 μL of “Protein Precipitation Solution” (Promega Corporation) were added to the samples, followed by incubation on ice for 5 min. Samples were then centrifuged (15,600×*g*) at 4 °C for 10 min. Supernatants were recovered and DNA was precipitated using 300 μL of isopropanol, followed by centrifugation (15,600×*g*) at 4 °C for 10 min. DNA precipitate was washed with 70 % ethanol and pelleted by centrifugation (15,600×*g*) at 4 °C for 10 min. DNA samples were then air-dried and dissolved at 4 °C overnight in 20μL of nuclease-free H_2_O. DNA concentrations and qualities were determined using an ND-1000 Spectrophotometer (NanoDrop Technologies, Wilmington, DE).

Plasmids from *E. coli* were isolated using the QIAprep Spin Miniprep Kit (QIAGEN Inc., Hilden, Germany). The same protocol was used for *S. aureus*, after treatment with lysis solution (0.5 μg/mL lysostaphin, 10 mM Tris–Cl, pH 7.5, and 1 mM EDTA).

Digestions with restriction enzymes (Promega AG, Dübendorf, Switzerland) were carried out according to the manufacturer’s specifications. PCR fragments were purified using the QIAquick PCR Purification Kit (Qiagen Inc.) and gel-bands were purified using QIAquick Gel Extraction Kit (Qiagen Inc.) according to manufacturer’s protocols. Ligations were performed using 1 μL of T4 ligase (Promega AG) according to the manufacturer’s specifications.

### PCR and quantitative PCR (qPCR)

All primers used in this study are listed in Table 2. GoTaq DNA polymerase (Promega AG) was used for colony PCR screening during cloning experiments and end-point PCR. DNA fragments required for cloning were amplified with KAPA HiFi DNA Polymerase (KAPA Biosystems, Cape Town, South Africa). All reactions were carried out according to manufacturers’ specifications.

**Table 2:**
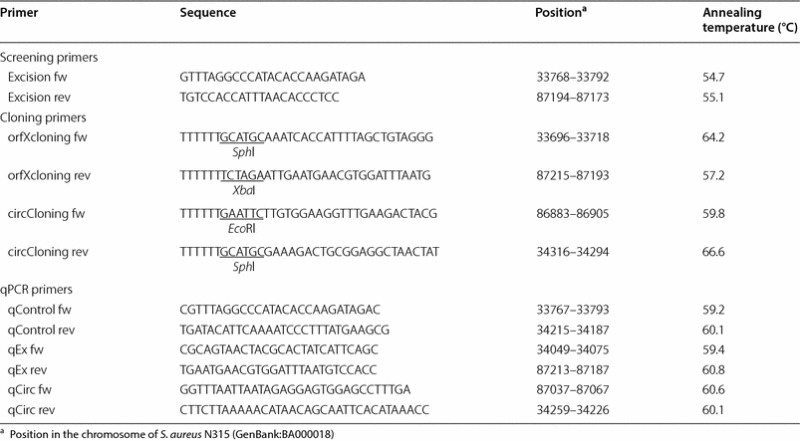
Primers used in the study

### Artificial excision of SCC*mec* to obtain N315EX

SCC*mec* was cured from *S. aureus* N315 to obtain N315EX using a method from Katayama et al. [7]. Briefly, strain N315 was electroporated with thermosensitive plasmid pSR3-1, containing the *ccrAB* genes and a tetracycline-resistance marker. Transformants were grown for 24 h at 30 °C in TSB supplemented with tetracycline, serially diluted and plated on TSA supplemented with tetracycline. Single colonies were picked, grown for 24 h at 42 °C TSB to promote cure of thermosensitive pSR3-1, and dilutions were plated on TSA to screen for colonies susceptible to both oxacillin and tetracycline. One double-susceptible colony, which lost both the plasmid (i.e. tetracycline-resistance) and kanamycin-resistance (encoded by SCC*mec*), was recovered and absence of SCC*mec* was confirmed by PCR amplification of qEx fragment using primers pair Excision fw and rev (Table 2).

### Co-culturing of N315 and N315EX

Co-culturing assays were performed in triplicates. 10^3^ CFU of overnight cultures of parent MRSA N315 and its excisant N315EX were washed with phosphate buffered saline, mixed at 10^2^ CFU/mL each in 10 mL of TSB, and incubated at 37 °C. The CFU counts of the two strains were determined by plating dilutions of the cultures at 0, 7, 24, 48 and 72 h. Strain N315 was selected for kanamycin-resistance (encoded by SCC*mec* and more reliable than resistance to oxacillin) on TSA plates supplemented with kanamycin, and total number of cells was determined by plating the competition mixture on TSA plates. Quantification of strain N315EX was calculated by subtracting the number of CFU on kanamycin plates from the number of CFU on plates without antibiotic.

### Designing a qPCR-based system to quantify site-specific excision of SCC*mec* and formation of the circular forms

**Fig. 1:**
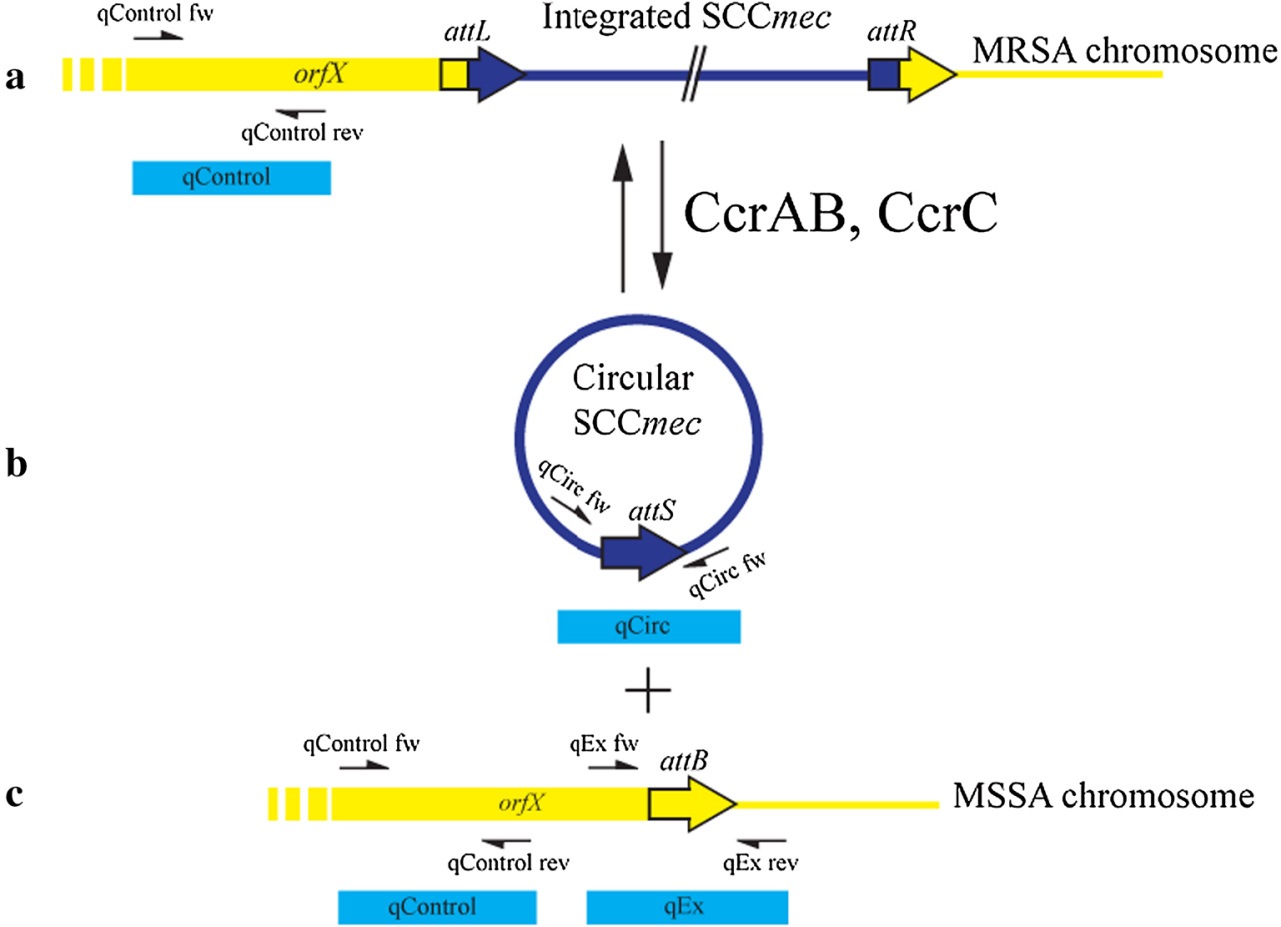
Schematic representation of SCC*mec* excision and formation of its circular form. The figure shows the linear SCC*mec* integrated in the chromosome (**a**), the circularized SCC*mec* excised from the chromosome (**b**) and the religated chromosome after excision (**c**). Chromosomal DNA is shown in *yellow* and SCC*mec* DNA is shown in *dark blue*. Locations of primers used for qPCR are shown by *arrows* and their amplicons are highlighted in *light* *blue*. qControl amplicon is used to determine the number of chromosomes in the assay, while qEx and qCirc specifically detect excisants and circularized SCC*mec*, respectively

Specific primers were designed using Primer3 software (version 2.3.4, [15]) and used to amplify the three targets depicted in Fig. 1. qControl amplicon, targets a region of *rlmH* gene, which is present in cells with and without SCC*mec*. This control was used to determine the number of total chromosomes in the samples. Amplicons qEx and qCirc were used to specifically detect excisants and circularized SCC*mec*, respectively. These were compared to the total number of chromosomes, which was determined by targeting a region of the *rlmH* gene, present in both MRSA- and MSSA-like cells.

In order to standardize the qPCR assay, qPlasmid, a plasmid carrying the three targets of the qPCR reactions (qCirc, qControl, and qEx) was constructed (Fig. 1). A 555-bp fragment containing the chromosomal junction formed upon excision, amplified from chromosomal DNA of *S. aureus* N315EX, was cloned in pUC28 using restrictions sites *Sph*I and *Xba*I. A second fragment of 411 bp, containing the *attS* site from the SCC*mec* circular form, was amplified from chromosomal DNA of *S. aureus* N315 and cloned using *Eco*RI and *Sph*I restriction sites.

Quant-iT™ PicoGreen^®^ dsDNA Assay Kit (Thermo Fisher Scientific AG, Switzerland) was used to determine concentration of qPlasmid, which was subsequently used to optimize the reactions efficiencies of qControl, qEx and qCirc amplicons. Different amounts of qPlasmid (40, 4 × 10^2^, 4 × 10^4^, 4 × 10^6^ and 4 × 10^8^ copies) were used as template for different primer concentration combinations in order to find the optimal conditions (Additional file 1: Supporting data 1).

qPCR reactions were performed in 20 μL volume reactions using the KAPA SYBR FAST qPCR Kit (Kapa Biosystems). DNA samples extracted from three independent cultures inoculated with 1 mL of overnight culture (approximately 10^9^ CFU) in 100 mL of TSB were used as template for the assays. When required, oxacillin was added at the concentration of 4 mg/L before adding the inocula. A total of 5 ng of DNA was used for each analysis. Reactions were carried out in MicroAmp optical tubes (Applied Biosystems, Foster City CA, USA) using an ABI7000 machine (Applied Biosystems). The following run protocol was used: initial denaturation step at 95 °C for 2 min then 40 cycles of 95 °C for 10 s and 60 °C for 40 s. Specificity of the qPCR amplification was confirmed by the sequencing of obtained DNA fragments (data not shown).

Standard curves prepared with different amounts of qPlasmid (40, 4 × 10^2^, 4 × 10^4^, 4 × 10^6^ and 4 × 10^8^ copies) were used to determine the number of copies in the samples using a linear regression model. All analyses were performed in triplicates.

### Determination of total chromosome copies (qControl amplicons), excised SCC*mec* (qEx amplicons) and circularized SCC*mec* (qCirc amplicons)

Genomic DNA samples were quantified using NanoDrop ND-1000 (Thermo Fisher Scientific AG) and diluted in nuclease-free H_2_O at a concentration of 10 ng/μL. A total of 10 ng was used for the qPCR assay. Quantification of qControl, qEx and qCirc amplicons was done by extrapolating the results from the standard curve obtained in the standardization experiments (see above), using the following formula:

$${\text{Sample copy number}} = 10^{{\left( {\left( {{\text{Ct}}_{\text{sample}} - b} \right)/a} \right)}}$$

where Ct is the threshold cycle; a and b are the slope and the y-intercept of the standard curve, respectively. The dynamic range of the assay ranged from 4 × 10^8^ to 40 amplicon copies. Proportions of excisants and circular form of SCC*mec* were calculated by dividing the absolute amount of the qEx and qCirc amplicons by the absolute amount of qControl amplicons. Negative controls consisting in nuclease-free H_2_O were included in the analysis.

### Statistical analysis

Unpaired *t* test was used to assess statistical differences between excision frequencies of cultures with different inocula (1/100 and 1/10,000). A *p* value of <0.05 was considered to indicate statistical significance.

## Results

### Rate of SCC*mec* excision in standard experimental conditions

Quantitative PCR was used to measure the amounts of both unoccupied chromosomal sites and SCC*mec* circular forms by specific amplification of the reconstituted chromosomal insertion site obtained after SCC*mec* excision and the junction formed upon SCC*mec* circularization, respectively (Fig. 1). These were compared to the total number of chromosomes, which was determined by targeting a region of the *rlmH* gene, present in both MRSA- and MSSA-like cells (Fig. 1).

Prior to the quantification of net excision rates, it was critical to ensure that the parent MRSA N315 and its excised mutant N315EX grew at the same rate in our experimental conditions. Any growth advantage of one of the strains would bias the results, since that strain would become overrepresented in the culture over time. Thus, a co-culturing assay in which 10^2^ CFU/mL of N315 and N315EX were inoculated together in 10 mL TSB was performed. Both strains grew at the same rate and did not interfere with each other for up to 72 h (Additional file 1: Supporting data 2).

**Fig. 2:**
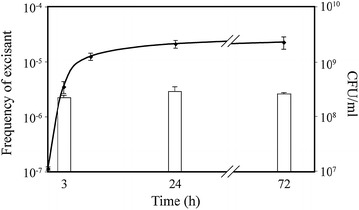
Growth curve and frequency of excisants in MRSA N315 grown in TSB. Bacteria were grown at 37 °C as described (*closed diamonds*) and sampled after 3, 24 and 72 h for DNA extraction and qPCR analysis. The *open columns* represent the ratio of excised versus chromosomes copies at each sampling time. *Error bars* represent the average ± SD of three independent measurements performed in three independent cultures

**Fig. 3:**
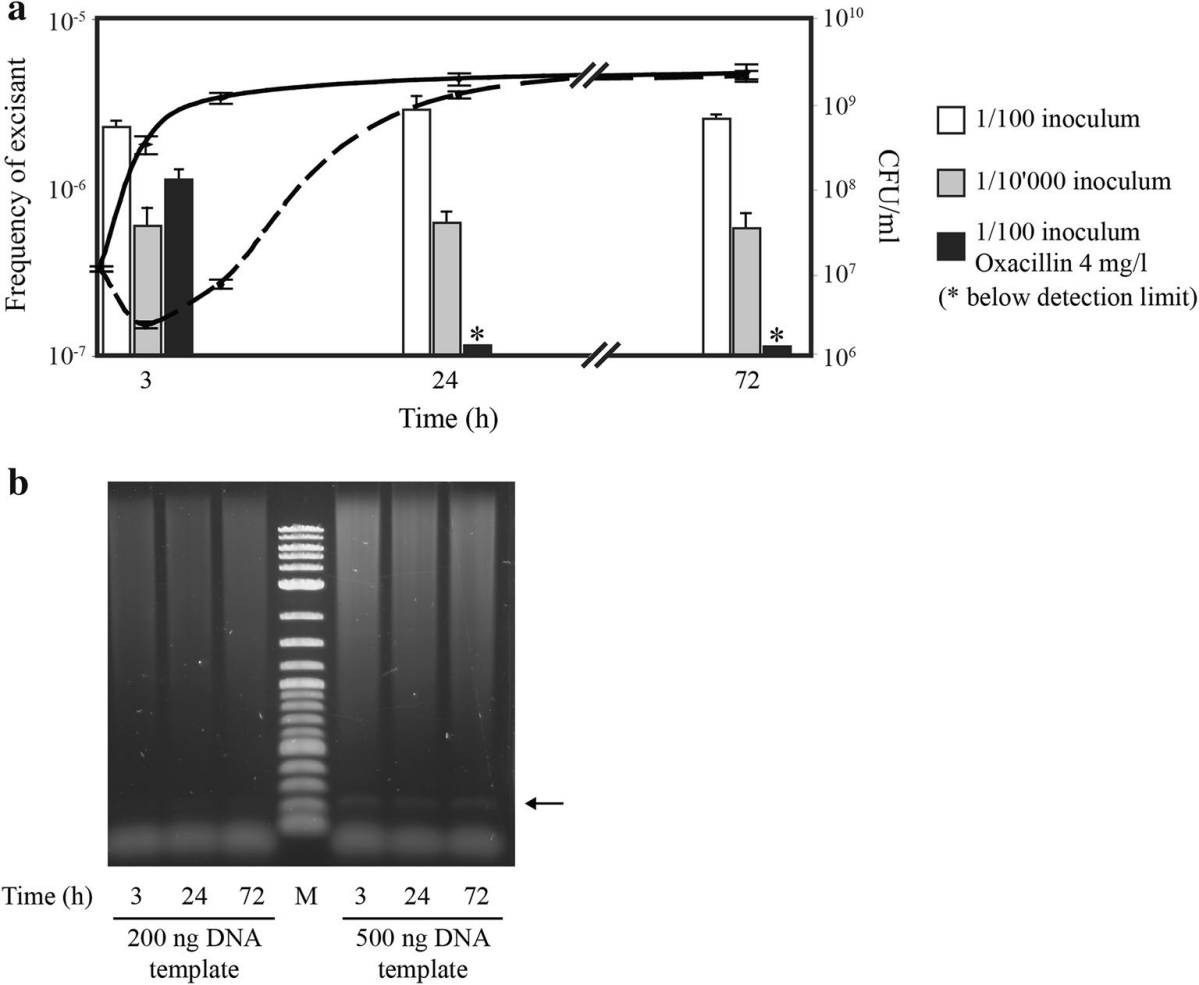
Determination of rates of excisants and presence of circular forms of SCC*mec* during growth of MRSA N315 various growth conditions. **a** In a first series of experiments, bacteria were grown at 37 °C in TSB (*continuous line*), but inoculated with either a 1/100 or a 1/10,000 dilutions of an overnight culture. *Open* *columns* represent the rates of excisants following inoculation with the 1/100 dilution and *grey columns* represent the rates of excisants following inoculation with the 1/10,000 dilution. In a second series of experiments oxacillin (final concentration of 4 mg/L) was added at the time of inoculation and both growth (*dotted line*) and rates of excisants (*black column*) were followed over time. *Asterisks* indicate that measurements were below the limit of detection. *Error bars* represent the average ± SD of three independent measurements performed in three independent cultures. **b** Presence of closed circular SCC*mec* during growth in TSB was detected by endpoint PCR using 200 or 500 ng of DNA previously used as template for qPCR. Circular SCC*mec* could not be quantified by qPCR because it remained below detection limits

The ratio of excised forms versus total chromosomes was first measured during growth at 37 °C in TSB. Figure 2 indicates that this ratio was approximately 2 × 10^−6^ already at first time point of the culture (i.e. 3 h), and remained constant in spite of further bacterial multiplication. However, our results also show that an overnight culture of N315 contains ca. 10^3^/mL excisants. In fact, such culture contains ca. 2 × 10^9^/ml cells and the frequency of excisants is 2 × 10^−6^/mL (Fig. 2). Knowing this, we noticed that when we were using a 1/100 dilution of an overnight culture as inoculum, 10 excisants/mL (i.e. MSSA cells) were readily introduced at beginning of the experiment. Therefore, our observations might result only from multiplication of the pre-introduced excisants. To answer this question, we performed the same experiment, except that we used a 1/10,000 dilution of an overnight culture as starting inocula. This ensured a theoretic carryover of less than one excisant (0.1 excisants/mL), in opposition to 10 excisants/mL inoculated with the 1/100 dilution. Figure 3 (grey columns) shows that a constant ratio of approximately 8 × 10^−7^ excisants was again present throughout the experiment, which could not result from carryover, indicating that excision occurred de novo. The significantly higher frequency of excisants observed for the 1/100 dilution inocula could be explained by the presence of the carryover from overnight cultures in addition to newly excised cells (Fig. 3).

**Fig. 4:**
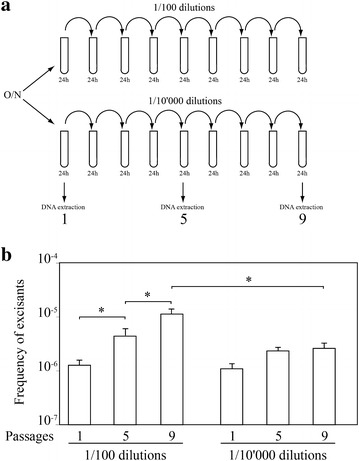
Determination of rates of excisants during serial subculturing of MRSA N315. **a** An overnight culture grown in TSB at 37 °C was used to inoculate a fresh 10 mL TSB culture with dilutions of 1/100 or 1/10,000. The new culture was allowed to grow overnight and used to reinoculate a new fresh culture, and this was repeated for 9 passages. DNA extractions and qPCR were performed at cycles 1, 5 and 9 as indicated. **b** Variation of the frequency of excisants over the passages. *Error bars* represent the average ± SD of three independent measurements performed in three independent cultures. Statistical differences were assessed by the unpaired *t* test and *asterisks* represent statistically significative results (*p* < 0.05)

To confirm our results, we tested the contribution of de novo excision to the total number or excisants in cultures undergoing successive passages. We reasoned that if de novo excision occurred in addition to carry over in new cultures, then repeating successive cultures should demonstrate a progressive accumulation of excisants, resulting from the sum of carry over plus de novo excisions. In contrast, if carry-over was the only source of excisants for new cultures, the frequency of 2 × 10^−6^ excisants should remain stable over time. A 1/100 dilution (i.e. 0.1 mL in 10 mL) from an overnight culture containing 10^9^ CFU/mL was inoculated into fresh broth, which was left to grow to the stationary phase (Fig. 4a). The new culture was grown to the stationary phase and used to reinoculate the next fresh culture, and the process was repeated for 9 consecutive passages. Figure 4b shows that the ratio of excised forms to total chromosomes increased approximately 10 times over the whole experiment. Thus, each of the 9 passages had contributed for an increase of a little more than 10^−6^ de novo excisants, which summed up to a tenfold increase in excisants (i.e. approximately 10^−5^ instead 10^−6^) at the end of the experiment. This experiment was also performed using 1/10,000 dilutions as inocula for consecutive cultures (Fig. 4a). The increase in excisant frequency was less pronounced and results were not significant as in the case of the 1/100 dilution.

### Detection of the circular form of SCC*mec* and effect of oxacillin treatment

In addition to the occupancy of the SCC*mec* insertion site, our study aimed at detecting the SCC*mec* circular form created upon excision. Extrachromosomal SCC*mec* cannot replicate autonomously and must be constantly generated *de novo* in order to be detectable. Using our experimental settings, quantification of the circular form remained below the limit of detection during all time-points. On the other hand, using endpoint PCR (40 cycles) with an excess of DNA template (approximately 0.5 µg), the specific junction formed upon SCC*mec* circularization could be detected at 3 h. Similar intensities of the bands suggested that approximately the same amount of PCR product was present at 24 h and 72 h, corroborating our hypothesis excision of SCC*mec* might take place only during the early stages of bacterial growth.

This hypothesis was further supported by the results obtained with oxacillin treatment. We reasoned that if excision occurred only transiently at the beginning of incubation and did not occur later in growth, then the oxacillin-susceptible excisants should be inhibited and become diluted off along growth. In contrast, if excisants would continuously arise at a sizeable rate (e.g. 2 × 10^−6^) from SCC*mec*-positive cells growing in oxacillin, then molecular signatures of de novo excised chromosomes should be detectable even if the resulting methicillin-susceptible excisants could not grow. Figure 3 shows that excisants rapidly disappeared after addition of oxacillin and that no detectable excision occurred thereafter. This supports the hypothesis that excision occurred transiently at the beginning of growth.

## Discussion

In the present study, we developed a qPCR-based method to monitor the rate of net excision of SCC*mec* in MRSA, taking strain N315 as model system. We found that in standard conditions, i.e. growth at 37 °C in TSB, the ratio of excised versus total chromosomes in N315 was approximately 2 × 10^−6^. This observation suggests that, in our experimental conditions, a culture of N315 is composed of a mixture of a majority of cells carrying SCC*mec* and of rare cells without it. This proportion was already present at the first checkpoint during growth, i.e. 3 h after inoculation, and remained constant until 72 h in the late stationary growth phase. The consecutive passage experiments further supported the finding that in the present experimental conditions, net excision occurred de novo at the rate of approximately 2 × 10^−6^. By diluting the inocula to 1/10,000, we excluded the possibility that detected excisants were due to carry-over from preceding cultures. We did observe that the excisants accumulated more slowly in the experiment with high 1/10,000 dilution, confirming that the frequency of excisants is the outcome of de novo excision and carry-over.

Net production of excisants in the presence of oxacillin was detected only during the early stages of growth, since the antibiotic was inhibiting the replication of bacteria without SCC*mec*, as expected. Several studies have assessed the expression of *ccrAB* genes in the presence of β-lactams [14, 16]. However, it is difficult to compare these results with our observations. First, our experiment measure net excision, and it is a net product of molecular process of excision and reinsertion, both depending on the activity of CcrAB recombinases. Moreover, the knowledge of post-transcriptional and post-translational regulation of CcrAB recombinases is quite limited. Nevertheless, differential expression of *ccrAB* genes between different MRSA strains could suggest that SCC*mec* excision might vary between different cassette types and MRSA backgrounds. Moreover, nucleotide variations in *ccrAB* genes from different SCC*mec* have been shown to influence the recombination activity and might influence the rates of excision as well [17]. Variation in the excision of SCC*mec* might be also observed in different ecological niches, as in the case of hospital-acquired MRSA and community-acquired MRSA, especially due to different antibiotic pressures. In the case of community-acquired MRSA, the smaller size of SCC*mec* might represent a factor facilitating the excision.

With our approach we measured net excision rates, and we could not directly determine how much actual excision and reinsertion occurred. However, the inability to detect the extrachromosomal circular form of SCC*mec* at all time-points suggested that excision might take place punctually during the early stages of growth. The consecutive passage experiments indicated that SCC*mec* is lost from cells during several cycles of replication, a situation that could theoretically lead to the fade of a given MRSA population. However, our experiment suggested several reasons why this may never happen. The most obvious is the selection pressure for maintenance of SCC*mec* and against excisants by antibiotics. Moreover, our experiments suggested that the excision happens mostly during the early growth of bacterial cells. The excision rate is likely much lower among MRSA colonizing human hosts, where the growth rates are certainly much slower. Additionally, because of their low frequency, the excisants might simply be extremely diluted or even lost during consecutive passages, e.g. from one human host to another, as suggested by the experiment with high 1/10,000 dilution, where the excisants accumulated more slowly than 1/100 dilution. These preliminary observations give important clues about the mobilization of SCC*mec*, a poorly understood process.

## Conclusions

Taken together, the present results indicate that excision of SCC*mec* occurred at a rate of approximately 2 × 10^−6^ in MRSA N315. This observation is concordant with a previous study in which the excision rate was evaluated to be lower than 10^−4^ [10]. The dynamics of the proportion of excisants during growth suggests that excision occurred rather early on, at a given cell density, and during a limited period of time. The technique described here for MRSA N315 is amenable to study excision of other types of SCC*mec* cassettes in other MRSA backgrounds, in order to better understand this crucial step of the horizontal transfer of this mobile element.

## Availability of supporting data

The data sets supporting the results of this article are included within the article (and its additional files).

## Additional file

10.1186/s13104-015-1815-3 Supplementary data 1 – qPCR reaction efficiencies.

## Abbreviations

SCC*mec*: staphylococcal cassette chromosome *mec*
MRSA: methicillin-resistant *Staphylococcus aureus*
PBP2a: penicillin-binding protein 2a
Ccr: cassette chromosome recombinases
LB: Luria–Bertani broth
TSB: trypticase soy broth
CFU: colony forming units
qPCR: quantitative PCR
SD: standard deviation
