# Supplementary material for: Excision of staphylococcal cassette chromosome mec in methicillin-resistant Staphylococcus aureus assessed by quantitative PCR
Source: BMC Res Notes. 2015 Dec 29;8:828. doi: 10.1186/s13104-015-1815-3 (PMC4693430; doi:10.1186/s13104-015-1815-3)
Supplement: Supplementary file 1 — 10.1186/s13104-015-1815-3 Supplementary data 1 – qPCR reaction efficiencies. [file 13104_2015_1815_MOESM1_ESM.docx]

**Supporting data 1**

| Primers concentrations  (nM) | Amplicon | | |
| --- | --- | --- | --- |
|  | qControl | qEx | qCirc |
| 50/50 | E = 97.52%  R^2^ = 0.9925 | E = 90.00%  R^2^ = 0.9951 | – |
| 75/75 | E = 97.55%  R^2^ = 0.9957 | E = 99.59%  R^2^ = 0.9983 | – |
| 100/100 | E = 110.57%  R^2^ = 0.9966 | E = 108.53%  R^2^ = 0.9972 | E = 98.49%  R^2^ = 0.9969 |
| 300/300 | E = 114.58%  R^2^ = 0.9988 | E = 111.19%  R^2^ = 0.9996 | E = 111.32%  R^2^ = 0.9993 |
| 900/900 | – | – | E = 112.99%  R^2^ = 0.9997 |

**Supporting data 1 – qPCR reaction efficiencies**

100-fold dilutions of qPlasmid were used as template for the three qPCR reactions in order to find the best primer concentrations (forward/reverse). “E” values represent the efficiency of the reaction and R^2^ values represent the correlation factor between replicates. Horizontal lines indicate that the reaction was not performed. Primer concentrations used in this study (highlighted in grey) matched the best amplification efficiency as well as correlation factor higher than 0.99.

**Supporting data 2**

**
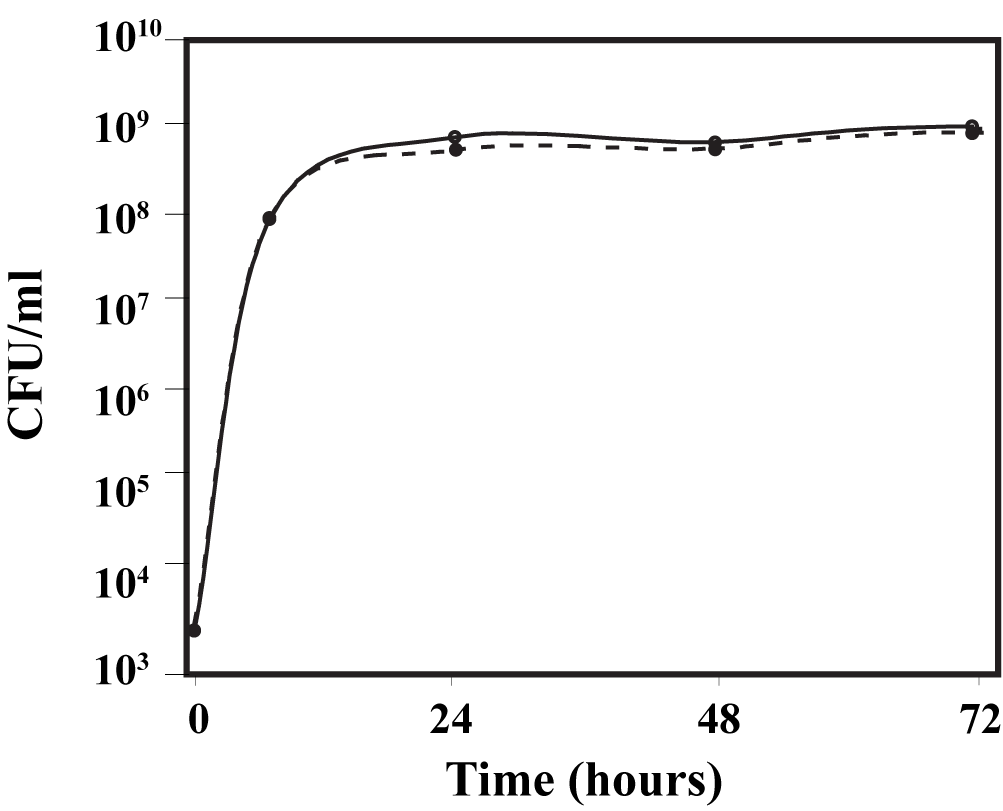
**

**Supporting data 2 – Fitness assay between N315 and N315EX**

Colony counts of the parent MRSA N315 (dotted line) and its excisant MSSA N315EX (black line) during growth in mixed culture. Samples were taken at various time points and plated on both plain agar and on agar containing kanamycin, which selected for the presence of SCC*mec*. The number or N315EX was calculated by subtracting the number of kanamycin-resistant colonies from the total number of colonies. The experiment was performed in triplicates.
